# Techno‐Functional Properties and Pharmaceutical Potential of Jackfruit Peel, Pulp, and Seeds

**DOI:** 10.1002/fsn3.71179

**Published:** 2025-11-09

**Authors:** Stéphano Tambo Téné, Donald Sévérin Dangang Bossi, François Zambou Ngoufack, Venkatachalapathy Natarajan

**Affiliations:** ^1^ Department of Biochemistry, Research Unit of Biochemistry of Medicinal Plants, Food Science and Nutrition, Faculty of Science University of Dschang Dschang Cameroon; ^2^ Department of Food Process Engineering National Institute of Food Technology, Entrepreneurship and Management Thanjavur Tamil Nadu India; ^3^ Department of Physiological Sciences and Biochemistry, Faculty of Medicine and Pharmaceutical Sciences University of Dschang Dschang Cameroon

**Keywords:** bioactive compounds, color, jackfruit peels pulp and seeds, rheological parameters

## Abstract

Jackfruit contains a significant quantity of secondary metabolites that are classified as high‐value biological compounds. The present work aimed to study the techno‐functional properties and volatile compounds in the peel, pulp, and seed of jackfruit. Color, amino acid composition, rheological parameters, determination of functional groups by FTIR, and determination of bioactive compounds by GC–MS were carried out. It was found that the values of a*, b*, and browning index were significantly (*p* < 0.05) higher in the peel compared to the values obtained from the seed and pulp. FTIR showed that the pulp had the highest number of peaks, followed by the seeds. A total of 6, 7, and 8 major amino acids were found in jackfruit peel, seed, and pulp, respectively. Lysine content was highest in all samples. The time–temperature relationship of gelatinization was highest in the seed, followed by the peel and then the pulp. Final viscosity was high in skin and seed compared to peak viscosity. A cumulative total of 136 compounds were detected in our three samples, including 67, 52, and 51 compounds in peel, seed, and pulp, respectively. Eight compounds, namely tetradecanoic acid, n‐hexadecanoic acid, octadecanoic acid, hexadecanoic acid, 2‐hydroxy‐1‐(hydroxymethyl)ethyl ester, butanoic acid, 3‐oxo‐,2‐methylpropyl ester,1,4‐dibutyl benzene‐1,4‐dicarboxylate, 4H‐pyran‐4‐one, 2,3‐dihydro‐3,5‐dihydroxy‐6‐methyl‐, and gamma‐sitosterol were present in different percentages in the three samples. The pulp appears to be the part with the most biologically active compounds, due to the greater number of peaks found by FTIR.

## Graphical abstract

FTIR showed that the pulp had the highest number of peaks. 8 volatile compounds were present in different percentages in the three samples.

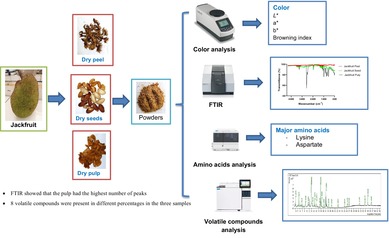

## 1 Introduction

Worldwide research into natural phytocompounds has expanded rapidly in recent decades due to their antioxidant, antidiabetic, antitumor, antimicrobial and anti‐inflammatory properties (Zahid et al. 2024). They are used in various fields such as pharmaceuticals, agri‐food, and cosmetics thanks to their strong antioxidant powers (Zahid et al. 2024). These bioactive compounds combat the increased production of free radicals, which are responsible for the degradation of biomolecules such as lipids, DNA, and proteins, leading to pathogenicity and chronic diseases such as cardiovascular disease, certain cancers, and diabetes (Durga et al. 2022). Thanks to their antioxidant powers, these compounds can simultaneously prevent and treat these diseases (Abu Bakar et al. 2015).

On the other hand, the use of synthetic antioxidants has often been criticized for causing side effects. It has been reported that the use of certain synthetic antioxidants in excess of recommended doses can cause adverse health effects, including tumors, mutations, and blood clotting problems (Rashid et al. 2022). To overcome this problem, researchers have turned their attention to the search for natural molecules in various biological matrices.

The rapid growth of the world's population has led to an increase in fruit and vegetable waste from industry, agriculture, and households. These wastes are most often skins, peels, seeds, and pits, which are potential sources of bioactive compounds such as carotenoids, polyphenols, terpenoids, saponins, alkaloids, tocopherols, etc. that have very high antioxidant properties (Manzoor et al. 2022; Saeed et al. 2022). Jackfruit (*Artocarpus heteroplyllus* Lam), also known as “poor man's fruit” by some, is a fruit of Asian origin found in abundance in tropical countries. Its pulp is highly prized by consumers, and its seeds have been increasingly used in human nutrition in recent years. It has been reported that jackfruit contains a significant amount of secondary metabolites that are classified as high‐value biological compounds, and the latter are thought to possess antioxidant, antimutagenic, and antiproliferative properties (Ruiz‐Montañez et al. 2015). In Cameroon, this fruit is still little known by most of the population, and even those who do know about it are interested only in the pulp. Its rich phytocompound content in the various parts of the plant has aroused the scientific curiosity of researchers in various countries. Several studies have been carried out on the leaves, roots, bark, pulp and seeds; but the fruit peel has not yet been the subject of many studies, and yet the peel of certain fruits, namely oranges (Dehghan et al. 2020), mangoes (del Pilar Sanchez‐Camargo et al. 2019), grapes (Sirohi et al. 2020), and pomegranates (Javani‐Seraji et al. 2023), have been studied and shown to be abundant in bioactive compounds such as phenols and flavonoids. Spectrophotometric analysis carried out by Abu Bakar et al. (2015) on extracts from the peels of three jackfruit species showed high antioxidant activity and phytochemical compound content compared with seed and pulp extracts. In addition to the presence of these numerous antioxidants, odorants, and antiproliferative molecules, studies have shown that this “waste” could be valorized in numerous formulations, given their functional potential and amino acid diversity (Amalia et al. 2020). In light of this previous work, the aim of the present project was to enhance the value of jackfruit peel by identifying the secondary metabolites responsible for the various biological activities in comparison with those of the pulp and seed.

## 2 Material and Methods

### 2.1 Source of Plant Material

Mature fruits were collected from a farmer in the locality of Bafou (Nkongni district, Menoua department, West Cameroon region) with geographical coordinates 5°28′00″ North, 10°07′00″ East and transported to the Research Unit of Biochemistry of Medicinal Plants, Food Science, and Nutrition of the Department of Biochemistry, Faculty of Science, University of Dschang (1500 m; 5°27′ North, 10°04′ East), where they were processed.

### 2.2 Methods

#### 2.2.1 Production Processes for Powders of the Various Fruit Parts

After transport to the laboratory, the fruit was washed and cut into 4 pieces, then the seeds, pulp, and skins were removed. Pulps and peels were blanched at 90°C for 10 min to prevent enzymatic browning. The various samples were dried in a ventilated oven (Venticell) at 45°C for 72 h, then ground using a blender (model MXAC2105, Panasonic, Japan). The powders were sieved (Ø = 350 μm), packed in plastic bags, and stored in a desiccator for future analysis.

#### 2.2.2 Determining the Color of Different Powders

The protocol described by Felisberto et al. (2015) was used to determine the colorimetric parameters of the various powders (CIELAB). To achieve this, 10 g of each powder was introduced into a glass vat so as to cover the surface of the base; then a flash from the Colorflex‐EZ device (A60‐1014‐593, Hunter Associates, USA) was used to evaluate the various parameters (L*, a*, b*). Equation (1) was used to determine the browning index:

(1) $$\text{Browning Index}=\frac{100\times \left(X-0.31\right)}{0.172}$$

with $X=\frac{a*+1.75L^{*}}{5.645L*+\left(a*-3.012b*\right)}$.

#### 2.2.3 Evaluation of Rheological Parameters of Different Powders

Powder rheology was carried out on a 15% aqueous suspension according to the protocol described by Sanchez et al. (2009). A Rapid ViscoAnalyzer model MCR 52 (Anton Paar) pre‐calibrated at 50°C was used to determine these parameters. The process began with stirring at 960 rpm for 10 s, followed by rotation at 160 rpm for the remainder of the process. Temperature evolution was marked by the following phases: holding at 50°C for 1 min and heating from 50°C to 95°C at a rate of 6°C.min^−1^, holding at 95°C for 5 min, cooling at a rate of −12°C.min^−1^ to 50°C, and finally holding at 50°C for 2 min. RHEOPLUS/32 Service V3.62 21,000,071–33,086 was used to analyze and extract the following parameters: pasting temperature (PT), pasting time (PTim), peak viscosity (PV: first peak viscosity following the pasting), holding viscosity (HS), and finally final viscosity (FV). Four additional parameters were then calculated: the breakdown (BD) (PV‐HS), the set‐back (SB) estimated by (FV—HS), the stability ratio (STR) (HV/PV), and the set‐back ratio (SBR) (FV/HV).

#### 2.2.4 Compound Profiling of Powders by FTIR (Fourier Transform Infrared Spectroscopy)

Determination of the functional groups present in the various powders was made possible by an IRAffinity‐1S Spectroscopy from SHIMADZU (Germany) using a Ceramic Rod IR energy source and a DLATGS detector. Starting with 0.3 g of powders placed on the FTIR's aluminum reading plate, the wavelengths of the spectra characteristic of the groups present were identified and recorded in the 400–4500 cm^−1^ range using Labsolution IR.

#### 2.2.5 Amino Acid Profiling and Quantification of Different Powders

Following digestion with 12 M HCl at 110°C for 17 h, the protocol described by Chinma et al. (2020) was used to profile and quantify amino acids. The resulting digest was filtered using a 0.45 μm millipore membrane, then the filtrates collected in vials were injected (1 μL at a flow rate of 1.5 mL/min) into an Ultra High Performance Liquid Chromatography (G7129B Agilent Technologies, Germany) coupled to a Diode Array Detector (UHPLC‐DAD). Amino acids were detected at 254 nm, and results were expressed in mmol/L based on a standard solution of 17 amino acids.

#### 2.2.6 Secondary Metabolite Profiling of Different Powders

Before determining the nature of the chemical compounds present in the various powders, the protocol described by Anibarro‐Ortega et al. (2020) was used to extract the phytochemical compounds. To achieve this, 1 g of each powder was mixed with 5 mL of MeOH (HPLC grade), vigorously vortexed (Borg Scientific, model LS3) at 90 rpm for 5, min and then transferred to a thermal shaker (Borg Scientific, model LS3) pre‐calibrated at 245 rpm at 37°C for 12 h. After stirring, the suspension was filtered through Whatman No. 4 paper containing 5 g Na_2_SO_4_ (soaked in MeOH). One (1) mL of extract was collected in vials (SIGMA‐ALDRICH Corporation, Bangalore, India) and transferred to an Agilent technologies Very High Resolution Gas Chromatography (Agilent 8890 GC System, Shanghai, China) coupled to a mass spectrometer (Agilent 5877B GC/MSD System) fitted with an Agilent 7693A automatic injector. The Rtx‐5MS column made of 5% diphenyl/95% dimethylpolysiloxane (30 m × 0.25 mm ID × 0.25 mm df) was used to separate phytochemical compounds. To achieve this, the temperature was calibrated at 250°C at a rate of 10°C/min starting from 50°C, for a final separation time of 40.50 min. The injector and mass spectrometer were calibrated at 280°C and 290°C, respectively. The injection flow rate was 1 mL/min for a helium transport speed of 25 cm/s for volatile compounds. Hexane was used as a cleaning solution, and 2 μL of each extract at a ratio of 10:1 was injected in split mode (60 psi) for 1 min at 250°C. The mass spectrometer calibrated at 250°C identified peaks at a voltage of 70 eV over a range from 50 to 550 AMU. The NIST 2020 library connected to the GC–MS post‐run analysis software Chrom Compare T1 (ChromSpace) installed on an hp. desktop (Agilent technologies) identified peaks, names, formulas, and retention times of the various compounds. Noise was completely eliminated during peak identification, and the percentage match between a peak and the proposed compound was 65%. The results are the average of 3 analyses.

### 2.3 Statistical Analysis

The results obtained are the average of three trials and were expressed as means ± standard deviations. Excel 2013 software was used for these calculations. Analysis of variance (ANOVA) using Minitab'R 18.1 software was used to compare the means, and Fisher's post hoc test was used to classify them at the 5% probability threshold.

## 3 Results and Discussion

### 3.1 Influence of Part on the Color of Different Samples

Color is an important parameter in the evaluation of the quality factor for the use of flours or powders in food formulations (Hadidi et al. 2020). The influence of the part on these different parameters can be explained by a variation in composition (Dangang et al. 2024). The value of a*, b*, and browning index (8.73 ± 0.01, 30.13, and 71.43 ± 0.03, respectively) was significantly (*p* < 0.05) higher in the peel compared to the values obtained from the seed and pulp (1.71 ± 0.01 and 6.25 ± 0.02, 16.97 ± 0.01 and 26.13 ± 0.02, 24.34 ± 0.02 and 58.57 ± 0.03 respectively) (Table 1). This would be due to the richness of phenolic compounds in the peel, which facilitates the action of enzymatic browning enzymes, resulting in the formation of brown, red, and black polymers (Ji et al. 2021). The seed showed the lowest values for these three points but a significantly high value (*p* < 0.05) for luminosity (80.80 ± 0.01) compared with the other two samples. This can be explained by their high protein and starch content (Amalia et al. 2020; Ji et al. 2021). Indeed, Amalia et al. (2020) and Tambo et al. (2024) reported that the high starch content in certain products gave them a white color. The brightness results (L*) obtained are lower than those of Flores‐Jiménez et al. (2024) (84.42) from defatted guamuchil seed flour, Bala et al. (2020) (88.85) from grass pea flour, and Pellegrini et al. (2018) (86.24) from quinoa seed flour, but higher than those obtained by Wani et al. (2021) (71.91) from *Echinocloa crusgalli* L. seed flour and Qadir and Wani (2023) (60.39) from rice flour. For the redness parameter (a*), the values obtained with peel and pulp were high compared to those of Qadir and Wani (2023) with rice flour (3.91 for a* and 22.15 for b*).

**TABLE 1:** Influence of fruit part on powder color.

Parameters	Peel	Seed	Pulp
L*	63.78 ± 0.01^b^	80.80 ± 0.01^a^	63.34 ± 0.01^b^
a*	8.73 ± 0.01^a^	1.71 ± 0.01^c^	6.25 ± 0.02^b^
b*	30.13 ± 0.01^a^	16.97 ± 0.01^c^	26.13 ± 0.02^b^
Browning index	71.43 ± 0.03^a^	24.34 ± 0.02^c^	58.57 ± 0.03^b^

*Note:* Means ± standard deviations followed by the same letter in the same row indicate that differences are not significant (*p* > 0.05).

### 3.2 Influence of Plant Part on the FTIR Profile of Different Samples

**FIGURE 1:**
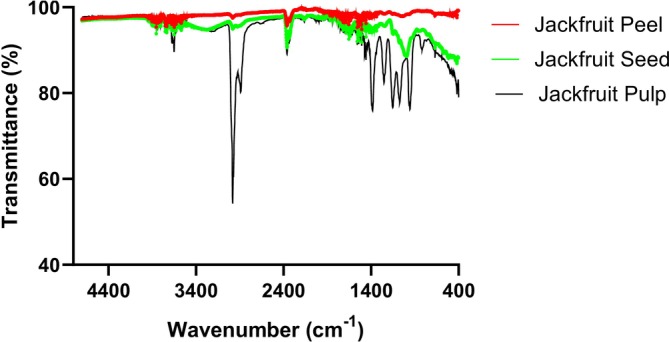
FTIR profile of different powders.

Figure 1 and Table 2 show, respectively, the FTIR profile of the different powders and the percentage formation of each compound peak. These show that the number of peaks, as well as the nature and percentage of each peak, varies with the sample. Indeed, pulp presented the greatest number of peaks, followed by seeds. This diversity of phytochemical compounds and their functional groups is thought to underlie the strong odoriferous properties of jackfruit pulp and may also be due to the numerous metabolic reactions taking place within it (Gunes and Karaca 2021). These observations are confirmed by the GC–MS profile, which revealed a high diversity of odorant compounds (aldehydes) in the pulp. Similar observations were also reported by Kohole et al. (2020) in various stabilized oils. Table 2 shows the vibration of peaks at transmittances ranging from 54% to 98% over a band from 4500 to 400 cm^−1^. In addition, there were 16 peaks in the peels, 25 peaks in the seeds, and 30 peaks in the pulp. The number of peaks identified does not match the GC–MS profile, which revealed more compounds in the skin. This can be explained by the fact that GC–MS allows compounds to be identified by name, whereas FTIR only reveals functional groups. However, according to Tambo and Natarajan (2025), several different compounds can have the same functional groups and therefore be identified as a single type by FTIR. It is also noted that 80% of the peaks identified in the three samples were at identical wavelengths, which would indicate the presence of similar compounds, although the variations in transmittance would reflect different concentrations. Tambo et al. (2022) report that the vibration of these peaks is the effect of different functional groups (‐CHO, ‐C = N, ‐CH3, ‐CH = CH‐, ‐COOH…) of molecules such as amino acids, fats, phenols, flavonoids, tannins, sugars, aldehydes, ketones, sulfur compounds… present in the different samples. Trans‐olefins marked by the presence of peaks between 700 and 400 cm^−1^ were identified only in pulp. Kohole et al. (2020) also identified such peaks at 722 cm^−1^ in peanut met as a result of caramelization or Maillard reactions. Two peaks materializing the presence of unsaturated hydrocarbons (1000 to 800 cm^−1^) were identified in pulp and seeds. This can be explained by the bleaching of the pulp, which would have been responsible for the degradation of the macromolecules leading to the production of these molecules (Kohole et al. 2020). Three peaks materializing the presence of primary and secondary alcohols and aromatic esters were identified at 1072.42, 1151.50, and 1251.80 cm^−1^ in the pulp. The presence of fermentative microorganisms in the pulp would be responsible for this observation. The presence of these aromatic esters is explained by photooxidation and thermal oxidation reactions. Similar observations and peaks were also identified by Lu et al. (2014). The presence of aliphatic‐chain amino acids in the various samples is materialized by the peaks at 1456.26 cm^−1^. The high percentage of this peak in peel is the effect of its glycine richness. Alanine concentration in pulp is shown by the presence of a peak at 1458.18 cm^−1^. The presence of unsaturated fatty acids, materialized by the vibration of the ‐CH groups of the methylenes, was identified at 1471.69 cm^−1^ in the peel and seed and at 1473.62 cm^−1^ in the pulp. These compounds were very intense in the peel, confirming the GC–MS results. Nitro‐rich compounds were identified in all samples between 1558.48 and 1506.41 cm^−1^. This would suggest high human activity in the area and that the samples were collected from fruit trees along asphalt roads. Nitro compounds are mainly found in automotive fuels and chemical pesticides. The structural organization of the proteins, in particular the amide II region, was materialized in the three samples by peaks between 1699.29 and 1635.64 cm^−1^. The presence of four peaks in this region in the seed is due to its high protein content (Dangang et al. 2024). Similarly, proline richness in these different samples, particularly in the seed, confirms β‐bends as the dominant secondary structure in proteins. Tambo et al. (2022) had also identified similar peaks in germinated cornmeal, while Aryee and Boye (2017) instead noted the presence of the characteristic β‐sheet vibrational groups at 1640 cm^−1^ in lentil proteins. These peaks are particularly intense in peel, and this would be the consequence of bleaching, which led to the destruction of other less stable secondary structures. Vibration of the carboxyl groups of amino acids and fatty acids was noted at 1716.65 cm^−1^ in the pulp and peel and at 1716.65 cm^−1^ then 1734.01 cm^−1^ in the seed. The results of GC–MS compound profiling show a high diversity of fatty acids in the seed. Similar observations were noted by Tambo et al. (2022) between 1440 and 1395 cm^−1^. The vibration of CO_2_ bonds is marked by the presence of peaks at 2358.94 and 2341.58 cm^−1^ in all three samples. The presence of two peaks in the pulp confirms the high fermentative activity of intrinsic microorganisms leading to alcohol production. The characteristic vibration of amine salts was identified at wavelengths 2889.37 and 2980.02 cm^−1^ in the pulp. These peaks are characteristic of the molecular interaction between macromolecules leading to the formation of new compounds such as amides and certain amino compounds. Lu et al. (2014) reported the presence of such compounds in fermented rapeseed oils. Between 3900 and 3566 cm^−1^, we note the presence of several peaks characteristic of the stretching of the ‐OH of free primary alcohols. These peaks are high in both seed and pulp. This may be linked to the high content of amino acids such as serine, reducing sugars such as mannopyranose, non‐reducing sugars like sucrose and starch in these two parts (Tambo et al. 2022).

**TABLE 2:** Influence of plant part on the number of FTIR peaks and the percentage of each peak.

Peel		Seed	Pulp
Peak (cm^−1^)	Area (%)	Area (%)	Area (%)
418.55			79.75
669.30			88.23
815.89			90.02
956.69			76.25
993.34		88.56	
1072.42			77.69
1151.50			76.62
1251.80			82.38
1456.26	96.26	93.06	94.56
1458.18			87.72
1471.69	97.08	94.51	
1473.62			88.50
1489.05	96.98	94.72	91.82
1506.41	94.84	91.89	91.00
1521.84			92.29
1539.20	95.52	92.20	
1541.12			91.44
1558.48	95.26	92.03	91.22
1635.64	96.85	93.03	93.34
1653.00	95.97	92.58	92.53
1683.86	96.41	93.97	93.81
1699.29		94.82	
1716.65	96.44	94.69	94.04
1734.01		95.33	
2341.58			91.40
2358.94	95.65	90.64	89.09
2889.37			80.21
2980.02			54.28
3566.38		94.64	95.30
3585.60		95.14	
3628.10	96.05	94.45	
3647.39	95.30	93.62	
3649.32			89.60
3687.90	96.44	95.24	
3709.11		95.23	
3711.04			95.49
3734.19	95.54	94.08	94.47
3749.62		94.31	
3801.70			95.81
3838.34		95.27	95.42
3851.85	95.35	93.84	
3853.77			94.98
3900.07		95.73	

### 3.3 Influence of Plant Part on the Amino Acid Profile of Different Samples

A total of 6, 7, and 8 amino acids were found in jackfruit peel, seed, and pulp, respectively (Table 3). This variation is in line with the work of Tambo et al. (2023), who also noted a difference in amino acid composition in sprouted corn grains. Similarly, the low diversity in amino acids can be explained by the extraction method and its sensitivity (Ma et al. 2019). We note the presence of two essential amino acids (histidine and lysine) in each sample, while in addition to serine, which is absent in the peel and seed, alanine is also absent in the peel. The low diversity of essential amino acids highlights the importance of diversifying protein sources when formulating foods including these plants. Lysine content was highest in all samples, particularly in seeds. This contradicts the findings of Tambo et al. (2022), who noted a loss of color in lysine‐rich corn flour. Indeed, the basic nature of this amino acid is thought to accelerate the non‐enzymatic browning reactions that can alter the color of samples. The change in whiteness observed in peel and pulp would therefore be due to the use of part of this amino acid in these reactions. Proline and then aspartic acid occupy second and third place, respectively, in terms of amino acid concentration. This is contrary to the data of Ma et al. (2019) and Perri et al. (2023), who respectively identified glutamic acid and leucine as the majority amino acids. Of the eight amino acids identified, five are soluble. These observations could explain the solubility of these powders in cold water. A look at the importance of the biological properties of these amino acids shows an absence of phenylalanine and tyrosine, suggesting that consumption of these powders would not lead to phenylketonuria but tyrosinemia, which can lead to goiter. These aromatic amino acids also have significant antioxidant and hepatoprotective properties. The absence of cysteine and methionine, which are essential for DNA synthesis and transcription, is due to their use in the plant's metabolic processes during growth. In addition to the absence of these few amino acids, Yeow et al. (2020) and Al‐Jibouri et al. (2016) reported that the high concentration of proline in these powders would be of great interest given its antioxidant capacity and its major role in the synthesis of phenolic compounds, notably coumarin and eugenol. Indeed, the metabolic pathway of proline biosynthesis is linked to that of erythrose‐4‐phosphate, which is the precursor of the shikimate pathway responsible for phenol synthesis (Shetty 2004).

**TABLE 3:** Amino acid composition of jackfruit peels, seeds, and pulp.

Parameters	Peel	Seed	Pulp
Essential amino acids (EAAs) (mmol/L)			
His	0.021	0.035	0.058
Lys	1.538	1.571	1.465
Non essential amino acids (NEAAs) (mmol/L)			
Ser	0.00	0.00	0.011
Gly	0.064	0.046	0.034
Asp	0.218	0.245	0.531
Glu	0.008	0.008	0.004
Ala	0.000	0.097	0.157
Pro	0.922	0.931	0.857

### 3.4 Influence of Plant Part on Rheological Parameters of Different Samples

Imoisi et al. (2024) suggest studying the rheological profile of a flour or foodstuff in order to determine its cooking profile and application conditions. Table 4 shows that the gelatinization time–temperature pairing was highest in the seed, followed by the peel and then the pulp. The jackfruit seed is very rigid in its wall, which would explain the use of a high temperature for a long time to weaken it and enable cooking. These observations are contrary to those of Pertiwi et al. (2022), who noted a drop in these two parameters in bleached starches. The fragility of the pulp could explain its gelatinization time of 5 min. In addition, the presence of amino acids such as aspartate in high concentration in the pulp would be responsible for its high gelatinization temperature, as it forms numerous solid bonds with other molecules of opposite charge through electrostatic interactions (Amalia et al. 2020). The results obtained by Amalia et al. (2020) on canistel nuts are more significant than those obtained in this study, confirming the value of certain heat treatments. During heat treatment, foodstuffs absorb water and swell. The viscosity measured during this phenomenon is known as peak viscosity (Tambo et al. 2024). This parameter ranged from 7037.00 cP (pulp) to 1184 cP (seed). The high fiber and soluble amino acid content of the pulp could explain these observations. Amalia et al. (2020) reported that the presence of ‐OH groups on the surface of soluble fibers facilitates good interaction with water. Similarly, bleaching of the pulp and peel would have resulted in a loss of soluble matter and weakening of the intermolecular bonds of the molecules present, leading to low water retention (Amalia et al. 2020). These results once again demonstrate the value of using pulp powder in formulations requiring good dough rise (Collado et al. 2011). Zhang and Hamaker (2012) obtained results superior to those of seed but less than those of pulp and peel. Good resistance to the effects of heat treatment is assessed by holding viscosity (Dongmo et al. 2020). To materialize this resistance, the variation between peak viscosity and holding viscosity must be close to 0. This parameter ranged from 3666.50 cP (pulp) to 507 cP (seed). This parameter corresponds to a variation of over 50% in the peak viscosity of seed and skin. This would testify to their low shear stability. Authors such as Dongmo et al. (2020) have reported that heat treatments such as boiling and blanching dissociate intermolecular fiber‐starch, protein‐starch, and lipid‐starch complexes, resulting in the loss of proteins and thus reducing the thermal stability of starch constituents. The data of Imoisi et al. (2024) are less significant than those of this study, confirming a weak association between the biological macromolecules of this plant. The viscosity reflecting the return to the polymeric state of certain molecules is materialized by the breakDown (Shafie et al. 2016). A high breakDown indicates a strong retrogradation. It varied between 3408.50 and 659.85 cP and was affected by the part of the plant. The high breakDown close to peak viscosity in peel and seed once again confirms the low thermal resistance of blanched foods. The values obtained were higher than those of Chung et al. (2009), confirming the strong molecular network present in cereals as opposed to fruit. The viscosity measured at the end of heat treatment is referred to as the final viscosity and provides information on the possible use of ingredients in the formulation of supplement foods (Malonado 2014). This final viscosity increased in the peel and seed compared to the viscosity at peak. Dongmo et al. (2020) explain this by the molecular reassociation of chemical macromolecules (proteins and carbohydrates) after bursting during heating. Indeed, the low molecular weight molecules released by hydrolysis during heating have low water retention and therefore low viscosity, unlike macromolecules. These results concur with those of Pertiwi et al. (2022), who noted an increase in the final viscosity of bleached canistel starch. Peel and seed powders could therefore be used in the production of jellies and the preservation of fresh foods. Amalia et al. (2020) also obtained less significant results than in this study in bleached and unbleached canistel nuts. During cooling, molecules that have retrograded have the ability to gel, and this is assessed by setback (Imoisi et al. 2024). It varied between 1052.20 and 5651 cP and was affected by the part of the plant. These results do not agree with those of Dongmo et al. (2020), who observed a reduction in setback in heat‐treated maize meal. They are consistent with those of Pertiwi et al. (2022), who noted an increase in setback after blanching canistel nuts. The values obtained by Pertiwi et al. (2022) are similar to those for seed but lower than those for peel and pulp. To assess the stability of the pastes formed during cooking of the different powders, setback and stability ratios were determined. Table 4 shows that pulp presented the best profile in terms of these two parameters, while peel powders proved the least stable during processing. It would therefore be important to combine both parts (peel and seed) of jackfruit with matrices rich in proteins and carbohydrates to ensure good stability.

**TABLE 4:** Rheological properties of different powders.

Parameters	Peel	Seed	Pulp
Peak time (s)	342.45 ± 10.54^b^	502.50 ± 10.61^a^	315.85 ± 5.87^b^
Pasting Temperature (°C)	50.47 ± 0.03^c^	55.93 ± 1.32^b^	72.67 ± 0.47^a^
Peak viscosity (cP)	3650.00 ± 67.88^b^	1184.00 ± 22.63^c^	7037.00 ± 2.83^a^
Holding viscosity (cP)	875.45 ± 6.43^b^	507.80 ± 1.13^c^	3666.50 ± 54.45^a^
Breakdown (cP)	2762.50 ± 44.55^b^	659.85 ± 0.21^c^	3408.50 ± 4.95^a^
Final viscosity (cP)	6526.50 ± 139.30^a^	1560.00 ± 45.25^c^	5116.00 ± 255.97^b^
Setback (cP)	5651.00 ± 145.66^a^	1052.20 ± 44.12^c^	1449.50 ± 310.42^b^
Setback ratio (FV/HV)	7.46 ± 0.21^a^	3.07 ± 0.08^b^	1.40 ± 0.09^c^
Stability ratio (HV/PV)	0.24 ± 0.003ᶜ	0.43 ± 0.009^b^	0.52 ± 0.008^a^

*Note:* Means ± standard deviations followed by the same letter in the same row indicate that differences are not significant (*p* > 0.05).

Abbreviations: FV, Final viscosity; HV, Holding viscosity; PV, Peak viscosity.

### 3.5 Influence of Plant Part on Volatile Compounds of Different Samples

**FIGURE 2:**
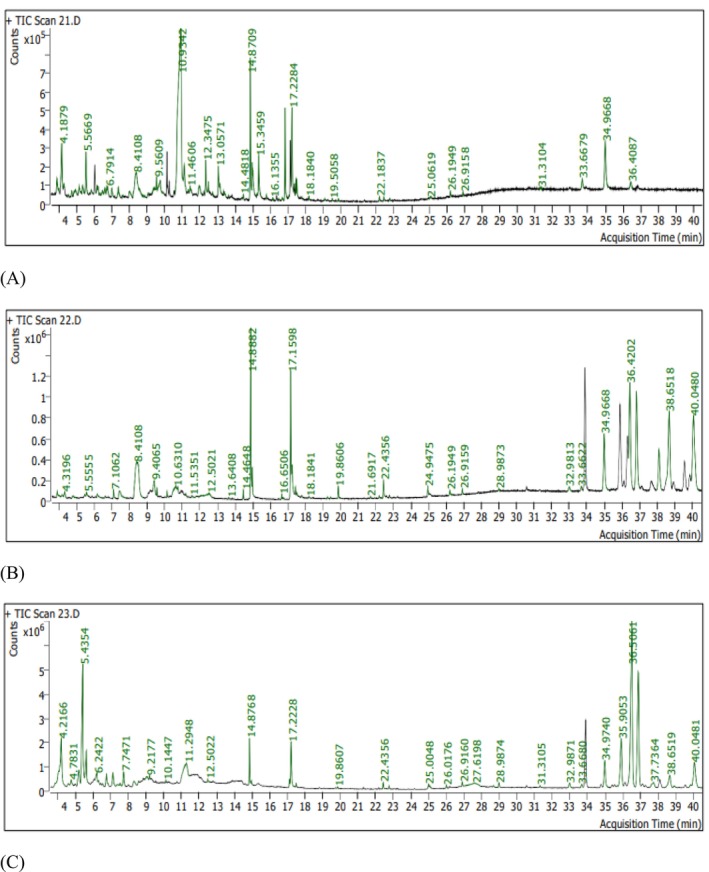
GC–MS chromatogram of peel (A), seed (B), and pulp (C) extracts.

Retention time, molecular formula, and area (%) of the compound identified by GC–MS (Figure 2) are presented in Table 5. A cumulative total of 136 compounds was detected in our three samples, including 67, 52, and 51 compounds in peel, seed, and pulp, respectively. Eight compounds, namely Tetradecanoic acid; n‐Hexadecanoic acid; Octadecanoic acid; Hexadecanoic acid, 2‐hydroxy‐1‐(hydroxymethyl)ethyl ester; Butanoic acid, 3‐oxo‐, 2‐methylpropyl ester; 1,4‐Dibutyl benzene‐1,4‐dicarboxylate; 4H‐Pyran‐4‐one, 2,3‐dihydro‐3,5‐dihydroxy‐6‐methyl‐; and gamma‐Sitosterol were present at different percentages in all three samples. The major compounds were n‐Butyric acid 2‐ethylhexyl ester (37.96%), d‐Glycero‐d‐ido‐heptose (6.64%), n‐Hexadecanoic acid (5.43%), 9,12,15‐Octadecatrienoic acid, (Z,Z,Z)‐ (5.23%), Gamma sitosterol (4.78%), and 9,12‐Octadecadienoic acid (Z,Z)‐ (3.59%) in the peel; 9,19‐Cyclolanost‐24‐en‐3‐ol, acetate, (3.beta.)‐ (13.23%), Lup‐20(29)‐en‐3‐one (11.98%), Lupeol (11.01%), Elemadienonic acid, methyl ester (10.11%), n‐Hexadecanoic acid (7.05%), 10E,12Z‐Octadecadienoic acid (6.13%), Gamma sitosterol (5.93%), and 24‐Methylenecycloartan‐3‐one (4.87%) in the seeds; Lup‐20(29)‐en‐3‐one (22.67%), Lupeol (14.35%), 5‐Hydroxymethylfurfural (11.11%), 4H‐Pyran‐4‐one, 2,3‐dihydro‐3,5‐dihydroxy‐6‐methyl‐ (6.26%), and Lanosterol (6.08%) in the pulp. It is important to note that some of these compounds, such as Hexadecanamide, n‐Hexadecanoic acid, 9,12‐Octadecadienoic acid (Z,Z)‐, 4H‐Pyran‐4‐one, 2,3‐dihydro‐3,5‐dihydroxy‐6‐methyl‐, 9‐Octadecanamide, (Z)‐ were also found in *Dendrobium* hybrid orchid and corn meal (Yeow et al. 2020; Tambo and Natarajan 2025).

**TABLE 5:** Volatile compounds.

No	RT (min)	Compound name	Molecular formula	Library MW (g/mol)	Area (%)			Match factor
					Peel	Seed	Pulp	
*Amides*								
1	6.3680	Octanamide, N‐(2‐mercaptoethyl)‐	C_10_H_21_NOS	203.10	0.10	nd	nd	56.60
2	17.7893	Hexadecanamide	C1_6_H_33_NO	255.30	nd	0.08	nd	66.70
3	19.2541	N‐[2‐(Dimethylamino)ethyl]‐2‐(phenylthio)acetamide	C_12_H_18_N_2_OS	238.10	nd	0.11	nd	60.50
4	26.1949	9‐Octadecanamide, (Z)‐	C_18_H_35_NO	281.30	0.25	0.40	nd	58.20
*Sugars*								
5	4.0679	D‐Mannopyranose	C_6_H_12_O_6_	180.10	nd	nd	2.43	74.90
6	6.6369	.beta.‐D‐Glucopyranose	C_6_H_12_O_6_	180.10	0.53	nd	nd	72.20
7	8.4108	Sucrose	C_12_H_22_O_11_	342.10	nd	10.82	nd	79.80
8	8.4108	d‐Glycero‐d‐ido‐heptose	C_7_H_14_O_7_	210.10	6.64	nd	nd	69.70
9	9.0860	5‐Thio‐D‐glucose	C_6_H_12_O_5_S	196.00	0.14	nd	nd	65.00
10	11.2948	Alpha‐l‐rhamnopyranose	C_6_H_12_O_5_	164.10	nd	nd	4.33	73.60
11	11.1287	Ethyl.alpha.‐d‐glucopyranoside	C_8_H_16_O_6_	208.10	2.10	nd	nd	76.60
12	27.6198	.alpha.‐D‐Glucopyranose, 4‐O‐.beta.‐D‐galactopyranosyl	C_12_H_22_O_11_	342.10	nd	nd	1.59	58.50
*Fatty Acids (FAs) and Fatty Acids Esters (FAEs)*								
13	4.3196	Hexanoic acid, 6‐hydroxy‐	C_6_H_12_O_3_	132.10	nd	0.45	nd	71.60
14	5.8759	Nonanoic acid	C_9_H_18_O_2_	158.10	0.27	nd	nd	72.20
15	6.5627	6‐Oxododecanedioic acid	C_12_H_20_O_5_	244.10	nd	nd	0.13	59.40
16	7.3980	n‐Decanoic acid	C_10_H_20_O_2_	172.10	0.69	nd	nd	84.90
17	10.1389	Dodecanoic acid	C_12_H_24_O_2_	200.20	nd	0.19	0.07	89.70
18	11.9756	Dodecanoic acid, 3‐hydroxy‐	C_12_H_24_O_3_	216.20	1.00	nd	nd	65.60
19	12.4963	Tetradecanoic acid	C_14_H_28_O_2_	228.20	0.33	1.94	0.13	86.60
20	14.4648	Hexadecanoic acid, methyl ester	C_17_H_34_O_2_	270.30	nd	0.36	nd	86.90
21	14.8709	n‐Hexadecanoic acid	C_16_H_32_O_2_	256.20	5.43	7.05	1.95	97.70
22	16.1355	Heptadecanoic acid	C_17_H_34_O_2_	270.30	0.13	nd	nd	65.10
23	16.4731	7‐Methyldecanoic acid	C_11_H_22_O_2_	186.20	0.08	nd	nd	59.80
24	16.6506	9,12‐Octadecadienoic acid, methyl ester	C_19_H_34_O_2_	294.30	nd	0.19	nd	87.30
25	16.7307	9‐Octadecenoic acid (Z)‐, methyl ester	C_19_H_36_O_2_	296.30	nd	0.10	nd	74.80
26	16.8393	9, 12‐Octadecadienoic acid (Z,Z)‐	C_18_H_32_O_2_	280.20	3.59	nd	nd	92.20
27	17.1370	(9E,11E)‐Octadecadienoic acid	C_18_H_32_O_2_	280.20	nd	nd	0.43	95.90
28	17.1598	10E,12Z‐Octadecadienoic acid	C_18_H_32_O_2_	280.20	nd	6.13	nd	98.70
29	17.2228	cis‐Vaccenic acid	C_18_H_34_O_2_	282.30	nd	nd	3.17	96.70
30	17.2284	9, 12, 15‐Octadecatrienoic acid, (Z,Z,Z)‐	C_18_H_30_O_2_	278.20	5.23	1.91	nd	96.20
31	17.5031	Octadecanoic acid	C_18_H_36_O_2_	284.30	0.81	0.14	0.15	89.70
32	19.4315	Z‐8‐Methyl‐9‐tetradecenoic acid	C_15_H_28_O_2_	240.20	nd	0.10	nd	63.90
33	22.4298	Hexadecanoic acid, 2‐hydroxy‐1‐(hydroxymethyl)ethyl ester	C_19_H_38_O_4_	330.30	0.21	0.95	0.35	66.70
34	24.9475	9, 12‐Octadecadienoic acid (Z,Z)‐, 2, 3‐dihydroxypropyl ester	C_21_H_38_O_4_	354.30	nd	0.66	nd	85.60
35	25.0735	9, 12, 15‐Octadecatrienoic acid, 2, 3‐dihydroxypropyl ester, (Z,Z,Z)—	C_21_H_36_O_4_	352.30	nd	nd	0.10	72.90
36	28.9873	Hexanoic acid, 2,7‐dimethyloct‐7‐en‐5‐yn‐4‐yl ester	C_16_H_26_O_2_	250.20	nd	0.17	nd	52.90
*Organic Acids*								
37	7.0317	Butanedioic acid, 2‐hydroxy‐2‐methyl‐, (S)‐	C_5_H_8_O_5_	148.00	0.54	nd	1.10	77.30
38	8.3251	D‐Glucopyranuronic acid	C_6_H_10_O_7_	194.00	nd	nd	0.74	75.40
39	9.4465	Butanoic acid, 3‐oxo‐, 2‐methylpropyl ester	C_8_H_14_O_3_	158.10	1.00	1.16	0.32	63.30
40	10.6310	3‐Deoxy‐d‐mannoic lactone	C_6_H_10_O_5_	162.10	nd	1.90	nd	80.20
41	10.7397	Ureidosuccinic acid	C_5_H_8_N_2_O_5_	176.00	nd	0.37	nd	51.30
42	10.9342	n‐Butyric acid 2‐ethylhexyl ester	C_12_H_24_O_2_	200.20	37.96	nd	nd	77.00
43	11.2032	Succinic acid, 2‐(2‐chlorophenoxy)ethyl ethyl ester	C_14_H_17_ClO_5_	300.10	nd	0.08	nd	62.50
44	12.2331	.beta.‐(4‐Hydroxy‐3‐methoxyphenyl)propionic acid	C_10_H_12_O_4_	196.10	0.07	nd	nd	63.00
45	13.1486	Benzoic acid, 4‐hydroxy‐3,5‐dimethoxy‐	C_9_H_10_O_5_	198.10	0.36	nd	nd	81.80
46	21.6917	Ethanethioic acid, S‐[2‐(dimethylamino)ethyl] ester	C_6_H_13_NOS	147.10	nd	0.12	nd	57.10
*Alcohols and polyols*								
47	5.5669	1,2,3‐Propanetriol, 1‐acetate	C_5_H_10_O_4_	134.10	2.05	nd	2.58	74.20
48	6.1792	3‐Hexanol, 3,5‐dimethyl‐	C_8_H_18_O	130.10	nd	0.33	nd	71.00
49	6.4939	4‐Octanol, 4,7‐dimethyl‐	C_10_H_22_O	158.20	0.38	nd	nd	56.40
50	7.1062	Cyclohexanol, 2‐(dimethylamino)‐, cis‐	C_8_H_17_NO	143.10	nd	0.47	nd	76.90
51	9.7726	2,4:3,5‐Dimethylene‐l‐iditol	C_8_H_14_O_6_	206.10	2.21	nd	nd	63.50
52	15.3459	trans‐Sinapyl alcohol	C_11_H_14_O_4_	210.10	2.25	nd	nd	90.2
53	15.7808	9, 12‐Octadecadien‐1‐ol, (Z,Z)—	C_18_H_34_O	266.30	0.15	nd	nd	77.60
54	17.0625	5‐Isopropenyl‐2‐methyl‐7‐oxabicyclo[4.1.0]heptan‐2‐ol	C_10_H_16_O_2_	168.10	0.09	nd	nd	55.20
55	19.8607	Ethanol, 2‐(9‐octadecenyloxy)‐, (Z)‐	C_20_H_40_O_2_	312.30	nd	nd	0.09	80.50
56	22.7674	E‐2‐Tetradecen‐1‐ol	C_14_H_28_O	212.20	0.11	nd	nd	70.30
57	36.4087	1‐Heptatriacotanol	C_37_H_76_O	536.60	0.74	nd	nd	57.00
*Esters*								
58	6.7801	Ethyl propionylacetate	C_7_H_12_O_3_	144.10	nd	nd	0.88	71.70
59	14.9797	1,4‐Dibutyl benzene‐1,4‐dicarboxylate	C_16_H_22_O_4_	278.20	1.07	1.10	0.20	92.20
60	17.4288	Isopropyl palmitate	C_19_H3_8_O_2_	298.30	nd	0.25	nd	67.50
61	18.1841	11‐Dodecyn‐1‐ol acetate	C_14_H_24_O_2_	224.20	nd	0.10	nd	72.40
62	19.8663	Z‐6‐Pentadecen‐1‐ol acetate	C_17_H_32_O_2_	268.20	0.12	nd	nd	61.40
63	22.7732	Z‐10‐Methyl‐11‐tetradecen‐1‐ol propionate	C_18_H_34_O_2_	282.30	nd	0.08	nd	65.60
64	22.7675	(Z)‐14‐Tricosenyl formate	C_24_H_46_O_2_	366.40	nd	nd	0.15	83.10
65	25.0048	2,3‐Dihydroxypropyl elaidate	C_21_H_40_O_4_	356.30	nd	nd	0.25	89.30
*Amines related compounds*								
66	4.3309	Ethanamine, N‐ethyl‐N‐nitroso‐	C_4_H_10_N_2_O	102.10	0.21	nd	nd	66.90
67	4.6115	4‐Methylproline	C_6_H_11_NO_2_	129.10	nd	nd	0.17	71.30
68	5.4640	Methiopropamine	C_8_H_13_NS	155.10	nd	0.12	nd	62.90
69	6.2422	trans‐4‐Hydroxy‐L‐proline, N‐methoxycarbonyl‐methyl ester	C_8_H_13_NO_5_	203.10	nd	nd	0.95	70.30
70	6.7399	L‐Isoleucine, N‐(methoxycarbonyl)‐, methyl ester	C_9_H_17_NO_4_	203.10	0.41	nd	nd	64.10
71	7.4381	N‐8‐Guanidino‐spermidine	C_8_H_21_N_5_	187.20	nd	nd	0.25	63.70
72	13.6408	Imidazole, 2‐amino‐5‐[(2‐carboxy)vinyl]‐	C_6_H_7_N_3_O_2_	153.10	nd	0.07	nd	61.50
73	17.7949	Deoxyspergualin	C_17_H_37_N_7_O_3_	387.30	0.07	nd	nd	58.10
*Ketones*								
74	3.9190	Dihydroxyacetone	C_3_H_6_O_3_	90.03	1.18	0.45	nd	73.40
75	4.1879	4H‐Pyran‐4‐one, 2,3‐dihydro‐3,5‐dihydroxy‐6‐methyl‐	C_6_H_8_O_4_	144.00	2.87	0.25	6.26	96.60
76	4.5655	Azetidin‐2‐one 3,3‐dimethyl‐4‐(1‐aminoethyl)—	C_7_H_14_N_2_O	142.10	0.12	nd	nd	57.50
77	4.7601	4H‐Pyran‐4‐one, 3,5‐dihydroxy‐2‐methyl—	C_6_H_6_O_4_	142.00	0.26	nd	0.48	80.07
78	4.7831	2,4‐Hexanedione, 5,5‐dimethyl‐	C_8_H_14_O_2_	142.10	nd	0.23	nd	65.70
79	5.2123	2 (3H)‐Furanone, 5‐heptyldihydro‐	C_11_H_20_O_2_	184.10	nd	nd	1.83	75.40
80	5.5555	Ketone, methyl 2‐methyl‐1,3‐oxothiolan‐2‐yl	C_6_H_10_O_2_S	146.00	nd	0.22	nd	67.20
81	6.1963	5‐Methyl‐2‐hexanone oxime	C_7_H_15_NO	129.10	0.49	nd	nd	67.90
82	6.2593	Pentalene‐1,5‐dione, 3a‐ (2,2‐dimethoxy)ethyl‐ hexahydro	C_12_H_18_O_4_	226.10	0.35	nd	nd	55.90
83	6.3223	1‐(Methylthio)‐3‐pentanone	C_6_H_12_OS	132.10	nd	nd	0.17	61.20
84	6.6256	5‐Methyl‐2‐hexanone oxime	C_7_H_15_NO	129.10	nd	0.12	nd	64.60
85	7.5469	Methane, dipropoxy‐	C_7_H_16_O_2_	132.10	nd	nd	0.24	64.00
86	7.7471	1‐Octene, 3‐(methoxymethoxy)—	C_10_H_20_O_2_	172.10	nd	nd	1.13	70.50
87	13.3489	2‐Amino‐3,5,7,8‐tetrahydro‐4,6‐pteridinedione	C_6_H_7_N_5_O_2_	181.10	0.07	nd	nd	62.50
88	13.6350	Cyclopentanone, 2‐(2‐octenyl)‐	C_13_H_22_O	194.20	0.09	nd	nd	62.40
89	14.4818	7,9‐Di‐tert‐butyl‐1‐oxaspiro (4,5)deca‐6,9‐diene‐2,8‐dione	C_17_H_24_O_3_	276.20	0.23	nd	nd	81.30
90	15.0140	6‐Methoxy‐7‐(3‐methylbut‐2‐enoxy)chromen‐2‐one	C_15_H_16_O_4_	260.10	1.45	nd	nd	76.50
91	22.1837	2‐Piperidinone, N‐[4‐bromo‐n‐butyl]‐	C_9_H_16_BrNO	233.00	0.23	nd	nd	73.40
92	25.0619	Tricyclo[5.3.1.1(2,6)]dodecane‐11,12‐dione, (1.alpha., 2.beta., 6.beta., 7.alpha.)—	C_12_H_16_O_2_	192.10	0.17	nd	nd	51.40
*Phenols*								
93	4.9718	Catechol	C_6_H_6_O_2_	110	0.73	nd	nd	92.30
94	6.7914	Phenol, 5‐ethenyl‐2‐methoxy‐	C_9_H_10_O_2_	150.10	0.52	nd	nd	83.10
95	8.5826	4‐Vinylbenzene‐1,2‐diol	C_8_H_8_O_2_	136.10	nd	nd	0.10	68.70
96	9.2177	Opuntiol	C_7_H_8_O_4_	156.00	nd	nd	0.15	75.50
97	9.5609	2,4‐Di‐tert‐butylphenol	C_14_H_22_O	206.20	0.79	0.23	nd	92.20
98	11.3576	2,4‐Dimethoxythiophenol	C_8_H_10_O_2_S	170.00	0.10	nd	nd	69.80
99	12.3475	(E)‐4‐(3‐Hydroxyprop‐1‐en‐1‐yl)‐2‐methoxyphenol	C_10_H_12_O_3_	180.10	1.25	nd	nd	93.20
100	16.6791	Isofraxidin	C_11_H_10_O_5_	222.10	0.23	nd	nd	77.40
*Sulfur compounds*								
101	12.7309	Thiophene, 2‐isobutyl‐5‐isopentyl‐	C_13_H_22_S	210.10	0.10	nd	nd	62.10
102	13.4004	2H‐1‐Benzothiopyran, 2‐ethyl‐3,4‐dihydro‐	C_11_H_14_S	178.10	0.25	nd	nd	61.20
*Phytosterols, terpenes, terpenoids and steroids*								
103	11.5351	aR‐Turmerone	C_15_H_20_O	216.20	nd	0.14	nd	63.00
104	26.9158	Squalene	C_30_H_50_	410.40	0.13	nd	0.22	51.80
105	26.9159	Supraene	C_30_H_50_	410.40	nd	0.30	nd	70.80
106	28.9874	Oct‐5‐en‐2‐ol, 8‐(1,4,4a,5,6,7,8,8a‐octahydro‐2, 5, 5, 8a‐tetramethylnaphth‐1‐yl)‐6‐methyl—	C_23_H_40_O	332.30	nd	nd	0.29	72.70
107	32.9813	5‐Cholestene‐3‐ol, 24‐methyl‐	C_28_H_48_O	400.40	nd	0.45	nd	68.60
108	32.9871	Campesterol	C_28_H_48_O	400.40	nd	nd	0.53	90.80
109	33.6679	Stigmasterol	C_29_H_48_O	412.40	0.85	0.33	0.32	72.10
110	34.9668	Gamma sitosterol	C_29_H_50_O	414.40	4.78	5.93	3.07	93.60
111	35.6821	. beta.‐Amyrin	C_30_H_50_O	426.40	nd	nd	0.15	72.60
112	35.9053	Lanosterol	C_30_H_50_O	426.40	nd	nd	6.08	91.80
113	36.4202	Lup‐20 (29)‐en‐3‐one	C_30_H_48_O	424.40	nd	11.98	22.67	86.30
114	36.8036	Lupeol	C_30_H_50_O	426.40	nd	11.01	14.35	86.10
115	38.0796	24‐Methylenecycloartan‐3‐one	C_31_H_50_O	438.40	nd	4.87	1.02	87.30
116	38.6518	Elemadienonic acid, methyl ester	C_31_H_48_O_3_	468.40	nd	10.11	nd	83.00
117	38.6519	Lanosta‐8,24‐dien‐3‐ol, acetate, (3.beta.)‐	C_32_H_52_O_2_	468.40	nd	nd	2.12	91.40
118	38.9094	Lup‐20 (29)‐en‐3‐ol, acetate, (3.beta.)—	C_32_H_52_O_2_	468.40	nd	nd	0.22	68.00
119	39.8478	12‐Oleanen‐3‐yl acetate, (3.alpha.)—	C_32_H_52_O_2_	468.40	nd	nd	0.33	80.80
120	40.0480	9, 19‐Cyclolanost‐24‐en‐3‐ol, acetate, (3.beta.)—	C_32_H_52_O_2_	468.40	nd	13.23	4.49	96.90
*Vitamins*								
121	31.3104	Vitamin E	C_29_H_50_O_2_	430.40	0.18	nd	0.14	50.60
*Chloride related compounds*								
122	19.8606	9, 12‐Octadecadienoyl chloride, (Z,Z)‐	C_18_H_31_ClO	298.20	nd	0.63	nd	88.10
*Miscellaneous compounds*								
123	7.4266	2,4,4‐trimethylpentyl ethylphosphonofluoridate	C_10_H_22_FO_2_P	224.10	nd	1.44	nd	76.90
124	26.0176	4‐(Chloromethyl)‐7‐hydroxy‐2H‐chromen‐2‐one, tert‐butyldimethylsilyl ether	C_16_H_21_ClO_3_Si	324.10	nd	nd	0.17	62.90
*Aldehydes compounds*								
125	5.1893	Benzaldehyde, 2‐methyl—	C_8_H_8_O	120.10	0.61	nd	nd	60.20
126	5.3724	5‐Hydroxymethylfurfural	C_6_H_6_O_3_	126.00	0.57	nd	11.11	86.30
127	8.0560	Vanillin lactoside	C2_0_H_28_O_13_	476.20	0.76	nd	nd	69.70
128	11.4606	Benzaldehyde, 4‐hydroxy‐3,5‐dimethoxy‐	C_9_H_10_O_4_	182.10	0.54	nd	nd	66.40
129	13.0571	2‐Hydroxy‐5‐methylisophthalaldehyde	C_9_H_8_O_3_	164.00	1.59	nd	nd	86.40
130	15.2543	trans‐Sinapaldehyde	C_11_H_12_O_4_	208.10	0.10	nd	nd	68.60
131	18.1840	E‐11,13‐Tetradecadienal	C_14_H_24_O	208.20	0.17	nd	nd	68.70
132	19.0881	Spiro[2,4,5,6,7,7a‐hexahydro‐2‐oxo‐4,4,7a‐trimethylbenzofuran]‐7,2′‐(oxirane)	C_12_H_16_O_3_	208.10	0.09	nd	nd	57.30
133	19.5058	5‐Hydroxy‐3‐methyl‐1‐phenylpyrazole‐4‐carbaldehyde	C_11_H_10_N_2_O_2_	202.10	0.08	nd	nd	61.60
134	22.1838	E‐15‐Heptadecenal	C_17_H_32_O	252.20	nd	0.17	nd	75.20
*Others compounds*								
135	7.6268	1,3‐Dioxane‐5‐methanol, 4,5‐dimethyl‐	C_7_H_14_O_3_	146.10	0.10	nd	nd	59.20
136	17.4459	Benzene, 1,2,4‐tributyl‐	C_18_H_30_	246.20	0.72	nd	nd	74.50

Abbreviations: MW, molecular weight; nd, not detected; RT, retention time.

For this study and subsequent discussion, only substances that matched GC–MS library data by greater than atleast 50% were selected. Of these compounds, several had recognized biological activities. n‐Decanoic acid, for example, was recognized as possessing anticancer, anti‐inflammatory, and antimicrobial activities (Huang et al. 2011). Dodecanoic acid is used to manufacture many soaps and shampoos, and flavoring agents (PubChem CID 3893). Tetradecanoic acid is known to possess antibacterial, antioxidative (Dwivedi et al. 2012), lubricant, cosmetic, and cancer preventive (Duke's's 2020) properties. Hexadecanoic acid, methyl ester has been asserted to possess anti‐inflammatory, anti‐cancer, antioxidant, hypercholesterolemia, and hepatoprotective properties (Tyagi and Agarwal 2017; Adenike et al. 2019; Choudhary et al. 2019), as well as antifungal, antibacterial, antiarthritic, and anticoronary activities (Tyagi and Agarwal 2017). Aparna et al. (2012) revealed that n‐Hexadecanoic acid possesses anti‐inflammatory properties by providing active binding sites that are compatible with phospholipase, thereby inhibiting their effect and preventing inflammation. It also exhibits selective cytotoxicity towards leukemic cells of humans (Harada et al. 2002). This compound also possesses antioxidant and hypocholesterolemic activity (Mensah‐Agyei et al. 2020). Maria et al. (2011) and Ravi and Krishnan (2017) have established the anti‐tumor activity of 9,12‐Octadecadienoic acid, methyl ester, by its cytotoxicity effect against cancerous cells. This compound is derived from an unsaturated fatty acid that has potential as an antibacterial agent and antifungal activity (Tahir et al. 2018). 9,12‐Octadecadienoic acid (Z,Z)‐ also called linolenic acid, is an omega‐6‐essential fatty acid, used in the biosynthesis of prostaglandins and cell membranes (Mensah‐Agyei et al. 2020) and has hepatoprotective, anti‐inflammatory, anti‐arthritic, and anti‐histamine activities (Henry et al. 2002), as well as antimicrobial activity (Duke's's 2020). 10E,12Z‐Octadecadienoic acid also known as (10E, 12Z)‐octadeca‐10,12‐dienoic acid is a derivative of linoleic acid. (9E,11E)‐Octadecadienoic acid is also known as the octadeca‐9,11‐dienoic with 9‐trans, 11‐trans stereochemistry. In addition to being a bacterial xenobiotic metabolite, it also functions as an antineoplastic, anti‐inflammatory, antiatherogenic, apoptosis inducer, and human metabolite (Pubchem). According to Yang et al. (2015), Cis‐vaccenic acid is an oleic fatty acid isomer that, in animal research and in several human cell lines, has been demonstrated to affect immunological function and to have protective effects against atherosclerosis, cancer, obesity, and diabetes. The compound 9,12,15‐Octadecatrienoic acid, (Z,Z,Z)‐ is an omega 3 fatty acid. It has been reported by Praveenkumar et al. (2010) that this substance has anti‐inflammatory, antidiabetic, hypocholesterolemic, anticancer, antiviral, antibacterial, antioxidant, and therapeutic properties for eczema. According to Duru and Maduka (2021), octadecanoic acid has antiviral, anti‐inflammatory, 5‐reductase inhibitory, hypocholesterolemic, and propecia activities. 9,12‐Octadecadienoic acid (Z,Z)‐, 2,3‐dihydroxypropyl ester decreases cholesterol level and inflammation. Additionally, it is an arthritic, anti‐inflammatory, anti‐acne, anti‐eczema, and 5‐alpha reductase inhibitor (Sivaranjani et al. 2021). Shobana et al. (2009) have reported the antibacterial activity of 3‐Deoxy‐d‐mannoic lactone. Benzoic acid, 4‐hydroxy‐3,5‐dimethoxy‐ is one of the derivatives of p‐hydroxybenzoic acid that inhibit polymerization of sickle Hb (Gamaniel et al. 2000) and also exerts analgesic as well as anti‐inflammatory action (Manuja et al. 2013). Trans‐sinapyl alcohol has cytotoxic activity against human tumor cell lines (Zou et al. 2006). 2,3‐Dihydroxypropyl elaidate presents antipyretic, anticonvulsant, antibacterial, and analgesic effects (Ravi and Krishnan 2017). As a member of the flavonoid group, 4H‐Pyran‐4‐one, 2,3‐dihydro‐3,5‐dihydroxy‐6‐methyl‐ has a potent antioxidant (Yu et al. 2013), making it a great scavenger of free radicals. It has been established that this compound can stop the proliferation of cancer cells and cause apoptosis in human colon cancer cells (Colworth et al. 1999). By preventing nuclear factor kappa B (NF‐KB), a transcription factor linked to several facets of oncogenesis, from activating, 4H‐Pyran‐4‐one, 2,3‐dihydro‐3,5‐dihydroxy‐6‐methyl‐ exhibits its anticancer properties. In addition, DDMP has anti‐diabetic properties via inhibiting α‐glucosidase (Quan et al. 2003), which lowers postprandial hyperglycemia and delays the absorption of glucose by preventing starch from breaking down into its monomers. Catechol has been shown to have antifungal and antibacterial effects (Kocaҫalışkan et al. 2006). Phenol, 5‐ethenyl‐2‐methoxy‐ is a volatile compound that has shown anticancer, antimicrobial, and antioxidant activities (Alghamdi et al. 2023). 2,4‐Di‐tert‐butylphenol is known to have cytotoxicity in human cells and animals, insecticidal and nematicidal activities, and antimicrobial activity (Zhao et al. 2020). (E)‐4‐(3‐Hydroxyprop‐1‐en‐1‐yl)‐2‐methoxyphenol exhibits antimicrobial property (Duke's's 2020). Campesterol is a naturally occurring plant sterol; it is well known to have anti‐inflammatory properties and to inhibit some germs from growing. Campesterol has been demonstrated to compete with 3‐hydroxy‐3‐methylglutaryl coenzyme A (HMG CoA) reductase, hence inhibiting the formation of cholesterol (Choi et al. 2007). Gamma sitosterol is known to have antidiabetic, anti‐angiogenic, anti‐cancer, antimicrobial, anti‐inflammatory, anti‐diarrheal, and antiviral properties (Sivaranjani et al. 2021). Lanosterol and derivatives possess antimicrobial activity (Shingate et al. 2013). Lup‐20 (29)‐en‐3‐one is a derivative of lupeol, which has been shown to have anticancer and antimicrobial activities (Machado et al. 2018). According to Liu et al. (2021), lupeol is a common natural triterpenoid found in both medicinal plants and edible fruits and vegetables. It has a wide spectrum of pharmacological properties, including antimalarial, antioxidant, antihyperglycemic, antitumor, antiviral, cytotoxic, and anti‐inflammatory activities (Zhang et al. 2019). 24‐Methylenecycloartan‐3‐one is a polygonum plant that has been found to have anticancer potential and antibacterial activity (Othman et al. 2014). Lanosta‐8,24‐dien‐3‐ol, acetate, (3.beta.)‐ has been reported to have antimicrobial, anti‐amylase inhibitory, antidiabetic, antioxidant, anti‐inflammatory, antiviral, and antifungal activities (Arora and Meena 2016). 12‐Oleanen‐3‐yl acetate, (3.alpha.)‐ has an anti‐inflammatory effect (Ding et al. 2010). 9,12‐Octadecadienoyl chloride, (Z,Z)‐ exhibits anti‐androgenic activity (Sudha et al. 2013). 5‐Hydroxymethylfurfural is found in many foods and is produced when foods containing reducing sugar and amino acids undergo Maillard reaction and the breakdown of hexoses in heat and an acidic environment. This compound exhibits higher antiproliferative activity on human melanoma A375 cells and antioxidant activity (Zhao et al. 2013).

## 4 Conclusion

The values of a*, b*, and browning index were significantly higher in the peel, reflecting its richness in phenolic compounds. The peel proved to be the richest in compounds with potentially bioactive functional groups, as revealed by GC–MS. In contrast, the pulp had the highest amino acid content. Several volatile compounds with biological activities were found in the different parts of the fruit studied. This study demonstrated the richness of bioactive compounds in the various parts of the jackfruit. The peel is also rich in these compounds, as are the pulp and the seed, and could be the focus of future research in the quest for compounds with pharmaceutical applications, as it showed the highest number of compounds.

For future work, we plan to determine the anti‐nutrient content of jackfruit skins with a view to their direct use as an ingredient in formulations, and also to determine their nutritional composition.

## Author Contributions

**Stéphano Tambo Téné:** Funding acquisition, investigation, methodology, validation, formal analysis, data curation, writing – original draft, review and editing. **Donald Sévérin Dangang Bossi:** Conceptualization, methodology, validation, formal analysis, investigation, supervision, data curation, writing – review and editing. **François Zambou Ngoufack:** Conceptualization, validation, supervision, writing – review and editing. **Venkatachalapathy Nataranjan:** Visualization, validation, supervision, writing – review and editing.

## Conflicts of Interest

The authors declare no conflicts of interest.

## Data Availability

Data is provided within the manuscript.
